# The evolution of microtubule associated proteins – a reference proteomic perspective

**DOI:** 10.1186/s12864-022-08502-y

**Published:** 2022-04-06

**Authors:** Amy C. Gottschalk, Marco M. Hefti

**Affiliations:** 1 College of Liberal Arts and Sciences, University of Iowa, Iowa City, USA; 2 Department of Pathology, University of Iowa, 25 S Grand Ave, MRC-108A, Iowa City, IA 52240 USA

**Keywords:** Microtubule associated proteins, Brain, Evolution, Tau, Microtubules, Cytoskeleton, Proteomics

## Abstract

Microtubule associated proteins (MAPs), defined as proteins that bind microtubules but are not molecular motors or severing enzymes, play a key role in regulating microtubule stability in neurons. Existing studies of the evolutionary relationships between these proteins are limited to genomic data from a small number of species. We therefore used a large collection of publicly available reference-quality eukaryotic proteomes to carry out a phylogenetic analysis of microtubule associated proteins in both vertebrates and invertebrates. Complete or near-complete reference quality proteomes were obtained from Uniprot. Microtubule associated proteins were identified using InterProtScan, aligned using MUSCLE and then phylogenetic trees constructed using the WAG algorithm. We identified 889 proteins with tubulin binding domains, of which 663 were in eukaryotes, including 168 vertebrates and 64 invertebrates. The vertebrate proteins separated into three families, resembling human MAP 2, MAP4 and MAPT, respectively, while invertebrate MAPs clustered separately. We found significant variation in number of microtubule associated proteins and number of microtubule binding domains between taxa, with fish and mollusks having an unexpectedly high number of MAPs and binding domains, respectively. Our findings represent a novel analysis of the evolution of microtubule associated proteins based on publicly available proteomics data sets. We were able to confirm the phylogeny of MAPs identified based on more limited genomic analyses, and in addition, derived several novel insights on the structure and function of MAPs.

**Supplementary Information:**

The online version contains supplementary material available at 10.1186/s12864-022-08502-y.

## Introduction

Microtubule associated proteins (MAPs), defined as proteins that bind microtubules but are not molecular motors or severing enzymes, play a key role in regulating microtubule stability. This group of proteins is of particular interest because they play a critical role in maintaining cytoskeletal stability in neurons and other cells with complex three-dimensional structures. Microtubule associated protein mutations, such as doublecortin (DCX), MAP2, MAP1A, MAP1B, and tau lead to varying degrees of central nervous system malformation and cognitive impairment in humans and in animal models [1–9]. One member of the microtubule associated protein family, tau, is also of particular interest because it has the unique ability to form toxic soluble oligomers, propagate and cause neurodegeneration in Alzheimer disease and related dementias [10–13]. It is the only microtubule associated protein capable of this toxic gain-of-function and appears to do predominantly, but not exclusively, in humans [14–24].

The vertebrate microtubule associated protein family includes three related proteins, tau (MAPT), MAP2 and MAP4, which are thought, based on genomic studies, to be the product of two duplication events ancestral to the vertebrate lineage [25]. These conclusions are however based on analysis of a small number of, predominantly vertebrate, genomes. The largest analysis to date includes 43 species, with only 11 invertebrates, and does not include the critical cephalopod family, which represents an independently evolved clade with a large and complex central nervous system and behavioral repertoire. Publicly available databases, including Uniprot, now include complete or near-complete proteomes for more than one thousand distinct species, making protein-based searches for specific domains practical. Although less well curated than nucleic acid-based databases such as GenBank, they provide an under-utilized resource for comparative evolutionary studies on the protein level, which we seek to exploit in our reported work.

We therefore used a large collection of publicly available reference-quality eukaryotic proteomes to carry out a phylogenetic analysis of microtubule associated proteins in both vertebrates and invertebrates. Our analysis is based only on the assumption that a microtubule associated protein will contain a tubulin binding domain, without any other a priori assumptions about protein or gene structure and includes all available complete or near complete eukaryotic reference proteomes in Uniprot.

## Materials and methods

### Data sources

We downloaded all reference proteomes annotated as complete or near complete in the Uniprot repository (Release 2020_3, 17 June 2020). We chose to use reference proteomes rather than specific mass spectrometry-based studies due to the lack of large-scale multi-tissue mass spectrometry studies across the tree of life (see discussion). Since our particular interest was in the potential role of microtubule associated proteins in the central nervous system, we focused our analysis on animals, excluding all other kingdoms. In order to be able to correlate our findings with characteristics of individual species, we also excluded metaproteomes and any protein not assigned to a single, distinct species. Data on brain size and number of neurons were obtained from published sources (Supplemental Table S1 in Additional file 1) [26–30]. Taxon membership was determined by manual curation of the species list. Since brain-specific proteomes were not available for most species we sought to study, we were not able to characterize the anatomic specificity or localization of individuals proteins.

### Identification and alignment of microtubule associated proteins

Interproscan was used with default settings to map all PFAM protein domains in the proteomes downloaded from Uniprot. Microtubule binding proteins were identified using the Unix *fgrep* function to identify all proteins containing a PFAM tubulin binding domain (PFAM00418). We then aligned the identified proteins using the multiple sequence comparison by log-expectation (MUSCLE) algorithm within MEGA [31]. We selected this algorithm based on benchmarking studies showing that it outperforms ClustalW, an alternate multiple sequence alignment algorithm, in both speed and accuracy [32]. A maximum likelihood tree was created from this alignment within MEGA using the WAG model, gamma distributed with invariant sites, and partial deletion settings according to published guidelines [33]. Protein searches against reference genomes were done using UCSC genome browser BLAT against the Genome Reference Consortium Zebrafish Build 11 (danRer11) or BLAST against the Ensembl octopus reference genome for *O. bimaculoides* (Genome assembly: PRJNA270931) since the UCSC genome browser does not contain a suitable reference genome for this species.

### Data analysis

Downstream data analysis and plotting was done using MATLAB R2021b (Mathworks, Natick, MA). A *p*-value cutoff of 0.05 was used for statistical significance. All code used for analysis is in Additional files 2 and 3.

## Results

### The number of MAPs varies significantly between taxa

**Fig. 1:**
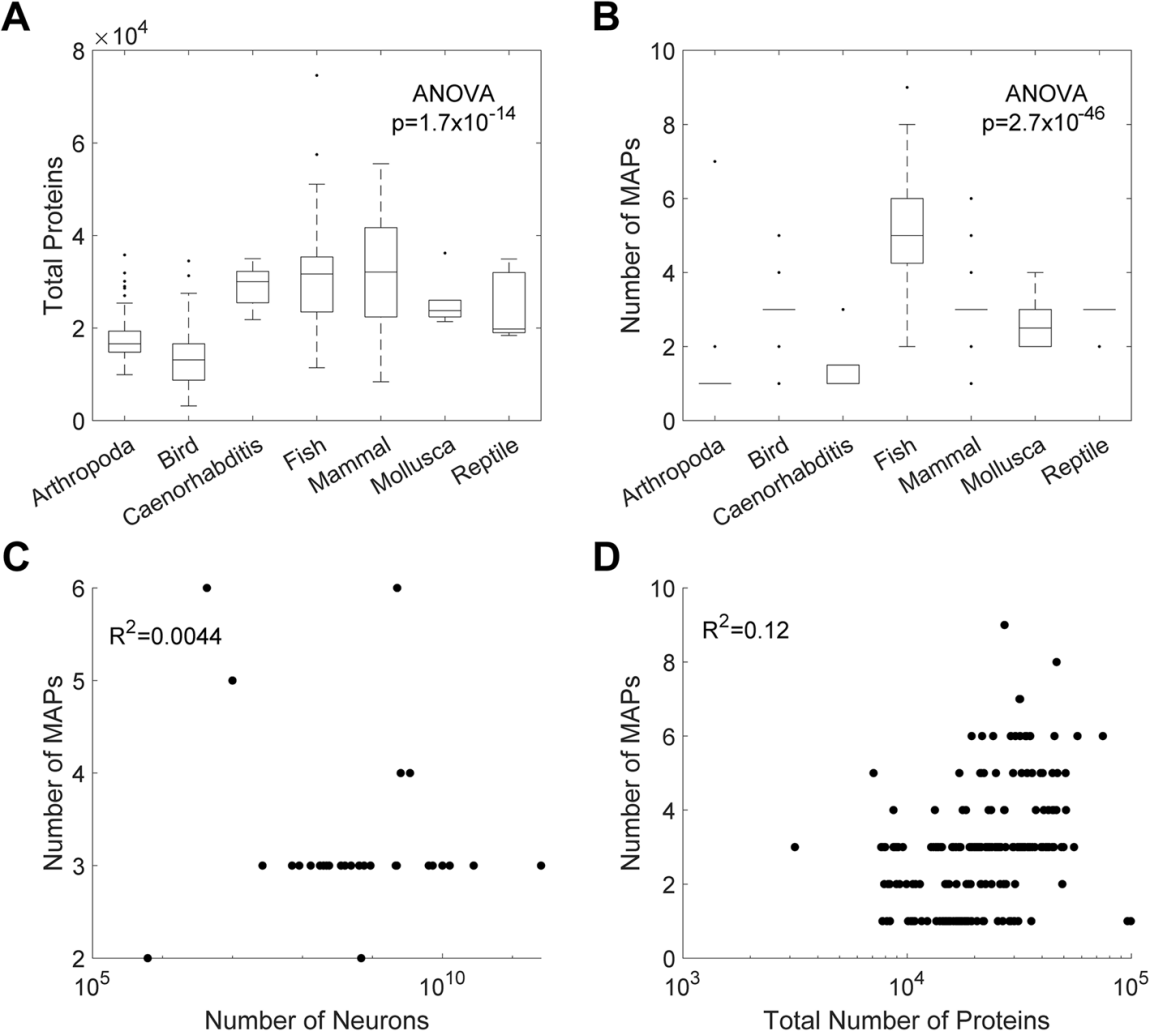
Number of microtubule associated proteins does not correlate with total protein number of brain size. Number of individual proteins (**a**) and microtubule associated proteins (**b**) in uniprot reference proteomes per taxa. Number of MAPs versus number of proteins (**c**) and number of neurons (**d**). ANOVA was used to calculate significance in panels (**a**) and (**b**) and Pearson’s correlation coefficient for R^2^ in (**c**) and (**d**)

**Fig. 2:**
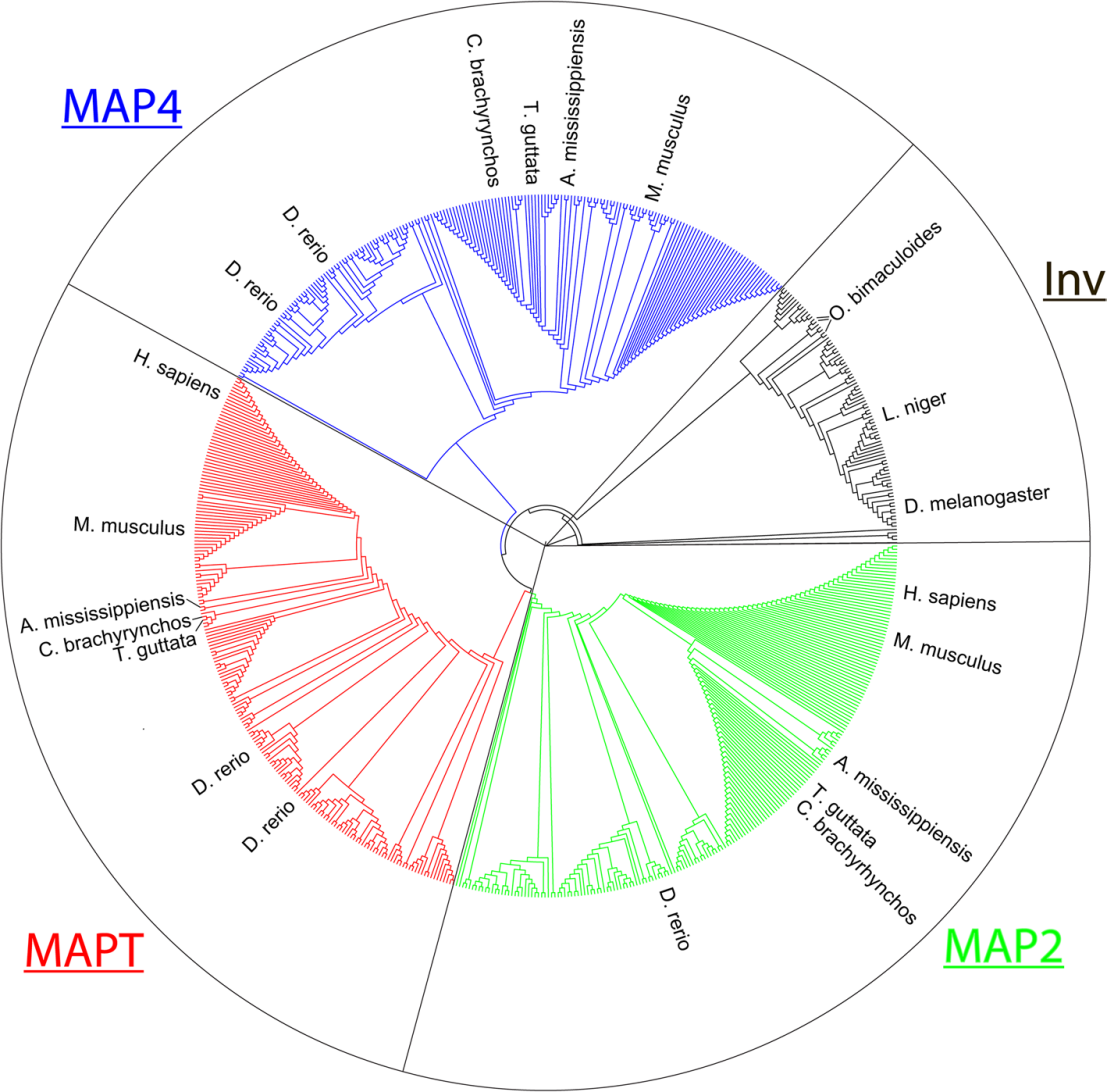
Phylogeny of microtubule associated proteins. Phylogenetic tree using showing all included species aligned using MUSCLE followed by tree construction using the WAG model, gamma distributed with invariant sites, and partial deletion settings according to published guidelines [33]

Of the 1478 species and 785,763,547 proteins initially investigated, we identified 889 proteins with tubulin binding domains. 663 of the MAPs belonged to a population of eukaryotic species which included 168 vertebrates and 64 invertebrates. The complete list of species and proteins is in Tables S2 and S3 (Additional file 1). This widely diverse group of eukaryotic species included mammals, birds, fish, reptiles, arthropods, nematodes, and mollusks. The median number of proteins per species varied significantly between taxa (mammals: 32100, birds: 13100, fish: 31700, reptiles: 19800, arthropods: 16600, nematodes: 30000, mollusks: 23750; Fig. 1A; ANOVA: *p* = 1.7 × 10^− 14^, Fig. 1A). Unexpectedly, fish have a significantly higher median number of MAPs per species when compared to all other taxonomic groups, with a median of 5 ± 1.49 proteins per species (Fig. 1B). This proliferation of MAPs does not appear to be a product of poorly annotated or fragmentary proteomes, as the identified proteins are not closely related (Fig. 2). In addition, when we examined zebrafish (*D. rerio*) proteins in more detail, we found that each identified MAP maps, with 100% homology, to a distinct gene in the zebrafish reference genome. Mammals, birds, mollusks, and reptiles had a median of 3 ± 0.73, 3 ± 0.75, 2.5 ± 0.82, and 3 ± 0.41 MAPs per species, respectively. Arthropods and nematodes had a median of 1 ± 0.88 and 1 ± 0.89, respectively.

### The number of MAPs does not vary significantly with proteome complexity or brain size

In order to rule out the possibility that variations in the number of MAPs were due to either a shift in genome complexity or a function of the completeness of individual proteomes, we then examined the relationship between total number of MAPs per species and neuron numbers. Neuron counts were derived from published sources as described in Methods above and in Additional file 1. Number of neurons explained less than 1% of variance in MAP number (R^2^ = 0.004, Fig. 1c). Similarly, the number of MAPs per species did not vary significantly with total protein number, suggesting that it is not a simple function of brain size or complexity (R^2^ = 0.12, Fig. 1D).

### Three microtubule associated protein families are shared by all vertebrates

We then aligned all MAPs identified by InterProScan using MEGA to generate a phylogenetic tree of all eukaryotic MAPs. This revealed three broad families corresponding to the human MAP2, MAP4 and MAPT (tau) proteins (Fig. 2). Birds, reptiles and mammals all showed the same set of three MAPs, with members of each family resembling each other more than they resembled other MAPs from the same species (Fig. 2). For example, the commonly studied zebra finch (*T. guttata*) and the North American crow (*C. brachyrhynchos*) both have close relatives of human MAPT, MAP2 and MAP4 (Fig. 2, outer). Interestingly however, the increased number of MAPs in fish appears to result from duplications within families, with the zebrafish (*d. rerio*) for example having two proteins clustering with mammalian tau (Fig. 2*, red*) and two with MAP4 (Fig. 2, blue) proteins. When we examined selected invertebrate species, the fruit fly (*d. melanogaster*) and black ant (*L. niger*) each showed one protein related to, but independent of, all three vertebrate clusters. Interestingly, the California two-spot octopus (*O. bimaculoides*) showed three closely related proteins all clustering with other invertebrates (Fig. 2, black). We then conducted a BLAT search to map these proteins back to original genes and found that all three are fragments of a single gene annotated as LOC106878541, with Gene ID: 106878541. It is not clear, based on the available data, whether these represent different isoforms of a single gene or different fragments of a single protein in this relatively understudied species.

### There is significant variation in the total number of microtubule binding domains per protein between taxa

**Fig. 3:**
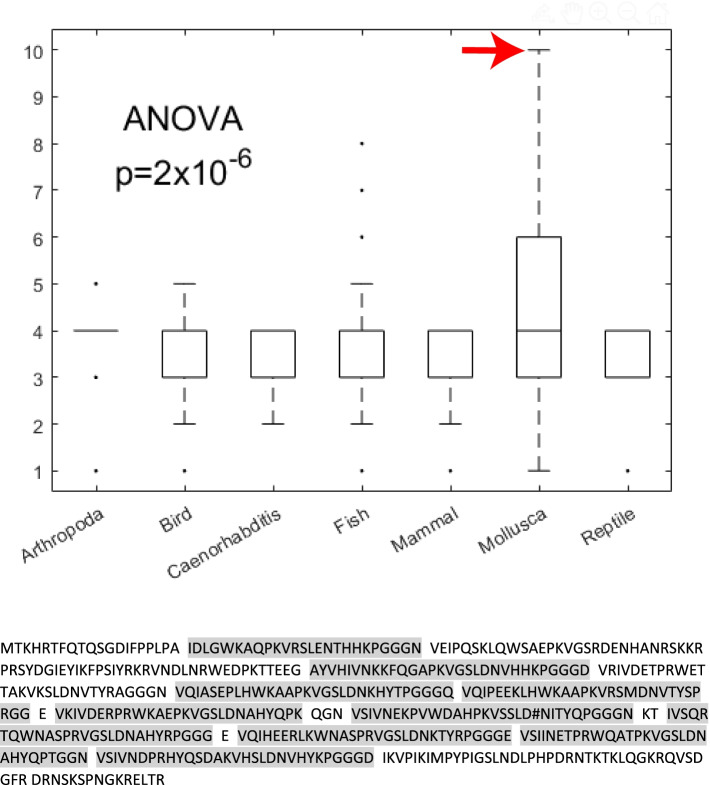
Variation in number of microtubule binding domains by taxon. Violin plot of MTBR’s per protein by taxa, with example of 10-repeat microtubule binding protein from Crassostrea gigas (pacific oyster), individual repeats are indicated by grey highlighting. Red arrow indicates position of sequence shown on boxplot

The number of microtubule binding regions per MAP is thought to be indicative of how well a protein binds to microtubules. The average number of microtubule binding regions of MAPs per taxon varied slightly (arthropods: 4, birds: 3, nematodes: 3, fish: 4, mammals: 4, mollusks: 5, reptiles: 3) (Fig. 3, top; ANOVA: *p* = 2 × 10^− 6^). Interestingly, mollusks have the highest average of microtubule binding regions with some mollusks possessing 7–10 (Fig. 3a). The pacific giant oyster (*C. gigas*), for example, has one microtubule associated protein with ten distinct microtubule binding domains (Fig. 3*, bottom*).

## Conclusions

In the current work, we constructed a systematic, unbiased phylogeny of vertebrate tubulin-binding microtubule associated proteins. As previously suspected based on genomic analyses, we found three distinct families of vertebrate microtubule associated proteins. While most vertebrate taxa (birds, mammals, reptiles) had one member of each family, fish showed additional expansions within each family and mollusks showed a remarkable expansion of microtubule binding repeats within proteins. We found no relationship between number of neurons or total number of proteins and number of microtubule associated proteins. Our findings show that the tau protein, which leads to neurodegeneration in Alzheimer disease and other neurodegenerative diseases of aging, is strongly evolutionarily conserved within the vertebrate lineage. The evolutionary origins of neurodegenerative disease, and neurodegenerative tauopathies specifically, thus remains unclear.

Our findings are broadly consistent with the phylogeny constructed by Sündermann et al. which is based on gene-level data on microtubule associated proteins [25]. Our work has several unique aspects which differ significantly from that of Sündermann et al., and lead to novel conclusions. First, we included a larger number of species (232 in our analysis versus 102 in Sündermann et al) and a far larger number invertebrates (64 versus 8). Our study includes mollusks, which were entirely absent from the previous analysis. This family is particularly important since it contains the cephalopods, which despite being invertebrates, have a complex central nervous system similar to that seen in mammals and birds. Second, our work focuses on protein-level data, potentially making it more sensitive to evolutionary relationships since amino acid sequences are more likely to be evolutionarily conserved than nucleic acids. Finally, our reported proliferation of microtubule binding proteins in fish, and the unexpectedly high number of microtubule binding repeats in mollusks is also unique and has not been previously reported.

Our findings are also consistent with the existing data on microtubule associated proteins in well studied model species showing one MAP in *c. elegans*, two in *d. melanogaster* and showing mouse homologues of all three proteins seen in humans. In addition, limited studies of MAPs in birds have shown that the domestic chicken (*g. gallus domesticus*) has 5 microtubule binding repeats, and we similarly identified five repeats in this species and in other related bird species [34]. Specifically, Uniprot A0A3Q2UDR8_CHICK has five distinct microtubule binding repeats. We also attempted to validate our data in a recent, large proteomic data set including 100 species across the tree of life [35]. Unfortunately, this dataset uses immortalized cell lines for all fifteen included eukaryotic species, most of which are of non-neuronal derivation. Examination of the data reveals that known brain-specific microtubule associated proteins (e.g., MAPT, MAP2) in humans or model species such as mice are represented, making comparison to our reference proteomic data impossible. This speaks to the need for systematic mass spectrometry-based studies of proteins from individual tissues, and particularly the nervous system, across the tree of life.

Although, thanks to the broad availability of eukaryotic proteomes we were able to examine a broad range of species across multiple taxa, adding validity to our results, we are limited by the organs/systems examined in each species. Microtubule associated proteins are best-known for their role in neuronal development and function, but they are expressed in other organs. The lack of brain-specific proteomes makes it difficult for us to determine whether the increased number of MAPs in fish or in the high number of MTBDs in mollusks reflects an increase in brain specific microtubule stability or reflects a greater need for microtubule stability in other organs, perhaps due to these species’ unique aquatic environment.

Given the enormous number of proteins studied, we were also not able to evaluate the evidence for each individual protein identified, instead relying on the curating efforts of Uniprot staff and contributors. Because we focused specifically on tubulin binding domains (our only a priori assumption for the purpose of this analysis) we did not identify the known human MAP’s MAP1a, MAP1b and MAP1c which do not contain classic tubulin binding domains studied in our current work. The microtubule binding domains in this family of proteins are less well annotated and show a lower degree of evolutionary conservation, making them difficult to identify in our analysis. We therefore cannot exclude the possibility that some of our studied species, particularly the less well-annotated invertebrates, have additional families of MAPs unique to them, which were not seen in our analysis. Ultimately, our analysis benefits from the breadth of data available in reference proteomes but is limited by the fact that these reference proteomes are derived from multiple studies with different tissues of origins, study objectives, and methods. This speaks to the urgent need for methodical proteomic studies of brain tissue across the tree of life, with a particular emphasis on understudied non-mammalian species with complex central nervous systems, such as birds and cephalopods.

The tau protein, which is the only microtubule associated protein capable of forming toxic aggregates in the aging brain, appears, based on our data, to be strongly evolutionarily conserved. It therefore remains unclear why it undergoes a toxic gain-of-function predominantly, but not exclusively, in humans [14–24]. Based on existing data showing early tau pathology in shorter lived animals such as cats, it is possible that the combination of tau, or a tau homologue, a large complex central nervous system and long lifespan are necessary to develop toxicity. Clarifying this question will require rigorous studies of aging, and particularly brain aging, in long-lived non-mammalian species (e.g., parrots).

Our findings represent a novel analysis of the evolution of microtubule associated proteins based on publicly available proteomics data sets. We were able to confirm the phylogeny of MAPs identified based on more limited genomic analyses, and in addition, derived several novel insights on the structure and function of MAPs. Our study is limited by the lack of brain-specific proteomes and limited proteome annotation for many species, underlying the importance of in-depth, tissue specific proteomic studies in non-model species. Future studies taking advantage of newly developed CRISPR protocols for non-model organisms, such as cephalopods, will be necessary to characterize the functions of individual MAPs and to understand the evolution of the apparent partial redundancy seen between mammalian MAPs [7–9, 36–38].

## Supplementary Information

**Additional file 1: Table S1.** References used for brain size and number of neurons. **Table S2.** Total number of MAPs and proteome size by species. **Table S3.** Microtubule associated proteins by species, taxon and binding regions.

**Additional file 2.** Linux bash script used for InterProtScan analysis. Code used to run InterProtScan on University of Iowa Argon HPC.

**Additional file 3.** Statistical analysis and graph generation. Code used for statistical analysis and generation of graphs.

## Data Availability

No novel datasets were generated as part of this study. All data used is publicly available via Uniprot (http://www.uniprot.org; RRID:SCR_002380) under the appropriate species identifiers. Code is included with supplementary materials.
